# Prevalence, trends, and correlates of HIV, syphilis, and HCV infections among male attendees at STD clinics in Southwest China: a 13-year cross-sectional study (2010–2022)

**DOI:** 10.1186/s12879-025-10571-9

**Published:** 2025-02-12

**Authors:** Chunxing Tao, Jianhua Che, Rongye Huang, Jinfeng He, Zhenxian Wu, Fengfeng Liang, Jie Cai, Yanyun Ou, Lijuan Bao, Li Ye, Hao Liang, Zhaosen Lin, Aidan Nong, Bingyu Liang

**Affiliations:** 1 Guangxi Key Laboratory of AIDS Prevention and Treatment, School of Public Health, Guangxi Medical University, Nanning, 530021 Guangxi China; 2 Chongzuo Center for Disease Control and Prevention, Chongzuo, 532200 Guangxi China; 3 Qinzhou Center for Disease Control and Prevention, Qinzhou, 535000 Guangxi China; 4 Collaborative Innovation Centre of Regenerative Medicine and Medical BioResource Development and Application Co-constructed by the Province and Ministry, Life Science Institute, Guangxi Medical University, Nanning, 530021 Guangxi China

**Keywords:** Male STD clinic attendees, Sexually transmitted infections, Trends, Risk factor

## Abstract

**Background:**

Sexually transmitted infections (STIs) impose a substantial health burden and pose a significant threat to human health. However, data regarding long-term epidemiology patterns of STIs among high-risk groups are scarce. This study aimed to evaluate the prevalence, trends, and correlates of HIV, syphilis, and HCV among male attendees at sexually transmitted disease (STD) clinics in Southwest China.

**Methods:**

Serial cross-sectional surveys were performed annually among male STD clinic attendees in Southwest China from 2010 to 2022. Blood specimens were collected to test HIV, syphilis, and HCV infections. Mann-Kendall trend test was used to assess the trends of HIV, syphilis, and HCV prevalence. Rare even logistic regression model (relogit) was used to identify correlates of HIV, syphilis, and HCV infections.

**Results:**

This study included a total of 23,964 male attendees at STD clinics. The prevalence of HIV, syphilis, and HCV among participants was 0.98%, 2.16%, and 0.61%, respectively. While the prevalence of syphilis and HCV decreased from 3.64% to 1.81% in 2010 to 1.05% and 0.38% in 2022, the HIV prevalence did not show a downward trend. Relogit analysis revealed that participants with a history of STD had significantly increased risks of HIV (aOR = 1.90, 95%CI: 1.14–3.15) and HCV (aOR = 4.91, 95%CI: 3.22–7.49) infections. Participants who had ever engaged in homosexual behavior had significantly increased risks of HIV (aOR = 14.66, 95%CI: 5.49–39.14) and syphilis (aOR = 3.95, 95%CI:1.41–13.71) infections. Age also played a role, with those aged 50 years and above having a higher likelihood of HIV infection (aOR = 2.55, 95%CI: 1.91–3.39), while those under 50 years were more likely to be infected with HCV (aOR = 1.94, 95%CI: 1.19–3.16). Moreover, individuals of Han ethnicity were more likely to be infected with syphilis (aOR = 2.12, 95%CI: 1.75–2.57) and HCV (aOR = 1.65, 95%CI: 1.16–2.33). Being married or cohabiting increased the likelihood of syphilis infection (aOR = 1.40, 95%CI: 1.09–1.80), and a history of intravenous drug use (IDU) significantly increased the risk of HCV infection (aOR = 10.97, 95%CI: 5.21–23.12).

**Conclusions:**

This study found a low prevalence of HIV, syphilis, and HCV among male attendees at STD clinics. Despite the declining prevalence of syphilis and HCV, HIV prevalence did not show a downward trend. This underscores the crucial need for continued and targeted prevention efforts, especially promoting STIs testing for men who have sex with men (MSM) and individuals with a history of intravenous drug use (IDU).

**Supplementary Information:**

The online version contains supplementary material available at 10.1186/s12879-025-10571-9.

## Introduction

Sexually transmitted infections (STIs) are infections that are transmitted through sexual contact. They are among the most common infectious diseases reported worldwide, with over one million new infections occurring daily [1]. The presence of STIs significantly elevates the risk of acquiring or transmitting human immunodeficiency virus (HIV) infection [1]. These infections substantially impact individuals’ quality of life, affecting them physically, psychologically, and socially [2]. Both globally and in China, STIs pose a growing public health concern, with increasing incidence rates in recent years [3].

Among the challenges posed by STIs, the co-occurrence of HIV, syphilis, and hepatitis C virus (HCV) infections is a particularly urgent issue. In China, HIV remains the top cause of mortality among notifiable infectious diseases from 2010 to 2019, with both its incidence and mortality rates on the rise [3]. HIV continues to be a significant global health challenge, with approximately 39 million people living with HIV (PLWH) worldwide [4]. HIV/AIDS is one of the most common incurable STIs, whereas syphilis, though a prevalent STI, is treatable [1]. However, PLWH are at an increased risk of syphilis infection [5]. Moreover, the World Health Organization (WHO) estimates that approximately 50 million individuals worldwide are chronically infected with HCV, a condition that can lead to cirrhosis or liver cancer [6].

While risk factors for STIs such as having multiple sexual partners, engaging in unprotected anal intercourse, and intravenous drug use have been studied [7], there is a lack of comprehensive data on the prevalence, trends, and factors associated with HIV, syphilis, and HCV among male attendees at STD clinics in Southwest China. STIs are known to impose significant health burdens [8]. In the case of Guangxi, a southern region in China, is facing one of the most rapid expansions of the HIV epidemic in the country. By October 2020, the cumulative number of individuals living with HIV in Guangxi had exceeded 97,000. Consequently, Guangxi ranks as the third-highest province in terms of reported HIV cases in China, accounting for 9.3% of the total cases despite representing less than 4.0% of the national population [9]. Guangxi’s unique geographic location, in close proximity to drug trafficking routes and sharing borders with Vietnam, adds complexity to the epidemiology of STIs. Cross-border mobility can significantly facilitate the spread of infections [10, 11]. Moreover, Guangxi has a high prevalence of injection drug use, a well-established risk factor for HIV and HCV transmission [12]. These factors create a dynamic environment that necessitates a dedicated investigation into the epidemiology of HIV, syphilis, and HCV. Guangxi is the only autonomous region in the southwest with border and coastal cities. Chongzuo City and Qinzhou City are in the southwestern region of Guangxi, China. Chongzuo City shares its border with Vietnam, an area known to host a substantial population of female sex workers (FSWs), many of whom originate from Vietnam and engage in commercial sex work [13]. Qinzhou City, as a coastal region in proximity to Vietnam, has a serious HIV epidemic and ranks third among Guangxi’s 14 cities in terms of cumulative HIV/AIDS cases [9].

Considerable evidence has been documented regarding the prevalence and associated risks of HIV/STDs in STD clinics [14–18]. However, prior investigations suffered from constrained sample sizes, and the data is outdated. A comprehensive dataset detailing recent temporal changes in HIV, syphilis, and HCV prevalence, along with associated risk factors among male attendees at STD clinics in Southwest China, is notably lacking.

Existing research tends to focus on specific populations, such as MSM [19–21] or FSWs [22–24], potentially missing a broader representation of those affected by STIs. Male STD clinic attendees, however, may act as the bridge that spreads HIV from high-risk groups to the general population. This study aims to bridge existing knowledge gaps by providing a comprehensive analysis of the epidemiological landscape of HIV, syphilis, and HCV infections among male attendees at STD clinics in Southwest China. We assessed the prevalence of these infections over the past thirteen years, identified temporal trends, and explored the sociodemographic and behavioral factors associated with them.

## Methods

### Study setting

From 2010 to 2022, we conducted a series of consecutive cross-sectional surveys as part of the routine sentinel STD surveillance program. This survey focused on male attendees at STD clinics in hospitals located at or above the county level in two cities (Chongzuo City and Qinzhou City), Guangxi. It took place annually from March to September.

### Study participants and procedures

Based on previous research, HIV had the lowest prevalence among the three STIs in this study. Therefore, the sample size was calculated using an estimated HIV prevalence of 0.7% among attendees in the STI clinics [17], ensuring sufficient statistical power to support all analyses for all three STIs. The calculation employed the Exact (Clopper-Pearson) confidence interval method with a two-sided interval, a 95% confidence level, and a confidence interval width of ± 0.5%. Using PASS (15.0.5) software, it was determined that each survey round would require 1,296 participants. This sample size is also adequate to detect the prevalence of other STIs, such as HCV and syphilis.

Participants for this study were recruited from STD clinics using convenience sampling. Clinical staff approached all male attendees presenting at the STD clinics during the study period in a systematic manner, regardless of their appointment status or reason for the visit. Eligible patients were screened consecutively as they arrived at the clinic and were invited to participate voluntarily if they met the inclusion criteria. This approach minimized selection bias and ensured that the sample represented male clinic attendees during the study period. Eligibility was determined based on the following criteria: (1) male sex; (2) aged at least 18 years; (3) visited an STD clinic because of concerns about a potential STI; and (4) willing to provide a written informed consent about the study.

Before the survey, participants were informed about the purpose of the survey. After obtaining written informed consent from participants, our trained CDC staff conducted face-to-face interviews in a private setting using a structured questionnaire. Each participant was assigned a unique identification number to ensure confidentiality and prevent duplicate registrations. Throughout the survey, our interviewers offered explanations and instructions to assist the participants in understanding the questionnaire.

Following the questionnaire survey, trained specialized staff collected blood samples from the participants for serological tests to determine the prevalence of HIV, HCV, and syphilis. The local Center for Disease Control and Prevention performed laboratory testing for HIV, HCV, and syphilis. Participants who tested positive for any of these infections were referred to the local treatment center for appropriate medical care.

### Socio-demographic, behavioral, and HIV-related knowledge and services variables

The survey collected socio-demographic data, including age, marital status, residency, and ethnicity. HIV-related knowledge regarding HIV transmission routes, treatment, and preventive measures was assessed using eight basic questions recommended by the Chinese CDC [25], which were widely used in China [26, 27]. Participants who scored 6 or higher on a scale from 0 to 8 on these questions were considered to have accurate HIV-related knowledge.

Behavioral variables were also measured by questions about various aspects of participants’ behavior. These questions included whether they had engaged in commercial sex with FSWs in the last three months, had engaged in sexual activity with a casual sexual partner in the last three months, had participated in homosexual behavior, had a history of STD, had a history of IDU, and whether they had received HIV-related services in the past year.

Participants self-reported if they had received HIV-related services in the past year. These services encompassed various aspects, such as free condom distribution, free HIV counselling and testing, free community drug maintenance therapy, clean needle provision or exchange, and free peer health education. Those who had received at least one of these services in the past year were considered to have received HIV-related services.

### Ethics statement

Each participant was compensated 20 RMB (about $3 USD) for participating in this study, which was approved by the Human Research Ethics Committee of Guangxi Medical University (Ethical Review No. 2013 − 130).

### Testing for HIV, syphilis, and HCV infections

Approximately 5 ml of peripheral blood was collected from each participant by trained physicians for serological testing. Briefly, blood specimens were tested for antibodies against HIV, syphilis, and HCV. Here’s a overview of the testing procedures:

1. HIV testing: The testing for HIV antibodies followed standardized operating procedures based on the manufacturer’s instructions. Initial screening for HIV involved using an enzyme-linked immunosorbent assay (ELISA) kit, HIV Ag/Ab ELISA - CE (Beijing Wantai Biological Pharmacy Enterprise, CO, Ltd.). Individuals who initially tested positive for HIV in the ELISA underwent a confirmation test using ELISA once again. Those who tested positive in the confirmation ELISA proceeded to a confirmatory test using the western blot (WB) assay, specifically the HIV blot 2.2 (Gene Lab Diagnostics, Singapore). A positive result in the WB assay confirmed HIV infection in the participant.
2. Syphilis Testing: Similar to HIV, initial screening for syphilis involved using an enzyme-linked immunosorbent assay (ELISA) from Wantai anti-TP ELISA (Wantai Biological Pharmaceutical Co. in Beijing, China). Individuals who tested positive in the initial screening underwent a confirmation test using the Syphilis Rapid Plasma Reagin (RPR) (Rongsheng Biotechnical Company, in Shanghai, China). Those who tested positive in the RPR confirmation were determined to be syphilis positive.
3. HCV Testing: HCV antibody testing was conducted using the AiD™ anti-HCV ELISA (Wantai Biological Pharmaceutical Co. in Beijing, China). Individuals who tested positive in the initial screening underwent a confirmation test, which confirmed the HCV infection.

### Statistical analysis

All data were double entered into EpiData version 3.1 software for Windows (Odense, Denmark) and then transferred to SPSS version 26.0 software (Chicago, IL, USA) and R version 4.3.1 for statistical analysis. The frequencies and percentages for qualitative variables were calculated by year of survey (2010–2022). Mann-Kendall trend test was used to assess the prevalence trends of HIV, syphilis, and HCV, as well as the trends in HIV-related knowledge, services, and behaviors. Rare even logistic regression model (relogit) was used to identify the factors associated with three binary outcome variables: HIV, syphilis, and HCV infections after testing for multicollinearity and excluding variables with variance inflation factor (VIF) > = 5 and correlation coefficient > = 0.8. As the three STIs were most commonly 0 and the standard deviation was larger than the mean [28], the analysis was performed using the Zelig package (https://github.com/IQSS/Zelig) in R. Crude odds ratios (cOR) from univariate logistic regression models were calculated, adjusted odds ratios (aOR) from relogit model and their respective 95% confidence intervals (95% CI) were presented in figures. All tests were two-sided, and the P-value < 0.05 was considered statistically significant. The figures were drawn using R, GraphPad Prism, and Photoshop software.

## Results

### Socio-demographic characteristics

As shown in Table 1, 23,964 participants enrolled in the study between 2010 and 2022. Most participants (71.98%, 17,249) were < 50 years old. About four-fifths of the participants (79.84%, 19,134) were married/cohabiting. The vast majority of the participants (92.81%, 22,242) were residing in Guangxi province. A significant proportion (62.56%, 14,993) of the participants belonged to national minority ethnic groups. During the study, increasing trends (P for trend < 0.001) were observed in the proportion of participants aged over 50 and belonging to minority ethnic groups. In contrast, there was a gradual decrease (P for trend < 0.001) in the number of participants with a marital status of married/cohabitating. Similarly, there was a decreasing trend (P for trend < 0.001) in the number of participants residing outside of Guangxi.

**Table 1:** Social-demographic of male STD clinics attendees recruited between 2010 and 2022 in Southwest China (n,%)

variable	Total	2010	2011	2012	2013	2014	2015	2016	2017	2018	2019	2020	2021	2022	cOR(95%CI)	*P* for trend
	*N* = 23,964	*N* = 1767	*N* = 2020	*N* = 2005	*N* = 2084	*N* = 2188	*N* = 2148	*N* = 2229	*N* = 1872	*N* = 1916	*N* = 1560	*N* = 1580	*N* = 1537	*N* = 1058		
**Age**																
< 50 years	17,249	1484	1557	1548	1647	1413	1562	1673	1292	1244	976	1120	1120	613	1.00	
	71.98%	83.98%	77.08%	77.21%	79.03%	64.58%	72.72%	75.06%	69.02%	64.93%	62.56%	70.89%	72.87%	57.94%		
>=50 years	6715	283	463	457	437	775	586	556	580	672	584	460	417	445	1.07(1.06–1.08)	**< 0.001**
	28.02%	16.02%	22.92%	22.79%	20.97%	35.42%	27.28%	24.94%	30.98%	35.07%	37.44%	29.11%	27.13%	42.06%		
**Marital status**																
Unmarried/widowed/divorced	4830	326	332	430	366	416	370	384	415	347	313	468	374	289	1.00	
	20.16%	18.45%	16.44%	21.45%	17.56%	19.01%	17.23%	17.23%	22.17%	18.11%	20.06%	29.62%	24.33%	27.32%		
Married/cohabitating	19,134	1441	1688	1575	1718	1772	1778	1845	1457	1569	1247	1112	1163	769	0.96(0.95–0.97)	**< 0.001**
	79.84%	81.55%	83.56%	78.55%	82.44%	80.99%	82.77%	82.77%	77.83%	81.89%	79.94%	70.38%	75.67%	72.68%		
**Residency**																
Guangxi	22,242	1586	1849	1882	1939	2033	2049	2032	1744	1770	1421	1455	1452	1030	1.00	
	92.81%	89.76%	91.53%	93.87%	93.04%	92.92%	95.39%	91.16%	93.16%	92.38%	91.09%	92.09%	94.47%	97.35%		
Outside Guangxi	1722	181	171	123	145	155	99	197	128	146	139	125	85	28	0.97(0.96–0.98)	**< 0.001**
	7.19%	10.24%	8.47%	6.13%	6.96%	7.08%	4.61%	8.84%	6.84%	7.62%	8.91%	7.91%	5.53%	2.65%		
**Ethnicity**																
Han	8971	658	752	658	894	876	931	860	817	799	520	530	518	158	1.00	
	37.44%	37.24%	37.23%	32.82%	42.90%	40.04%	43.34%	38.58%	43.64%	41.70%	33.33%	33.54%	33.70%	14.93%		
Other minorities	14,993	1109	1268	1347	1190	1312	1217	1369	1055	1117	1040	1050	1019	900	1.03(1.02–1.04)	**< 0.001**
	62.56%	62.76%	62.77%	67.18%	57.10%	59.96%	56.66%	61.42%	56.36%	58.30%	66.67%	66.46%	66.30%	85.07%		

Note: Statistically significant P values are indicated in bold

### Behavioral characteristics and HIV-related knowledge and services

Table 2 presents the characteristics of the 22,955 participants after excluding incomplete data. Among the participants, a substantial majority (93.32%, 21,422 participants) exhibited adequate knowledge about HIV. Approximately 29.56% (6,785 participants) reported having engaged in commercial sex with FSWs, while 24.71% (5,762 participants) had engaged in sexual encounters with casual sex partners within the preceding three months. A small proportion of participants reported a history of IDU (0.60%,138 participants), reported engaging in homosexual behavior (0.23%, 52 participants), and disclosed a history of STIs (5.09%, 1,169 participants). In terms of HIV-related services, the highest acceptability rate was for condom promotion and distribution or HIV/AIDS voluntary counseling and testing (65.69%, 15,078 participants). Conversely, the rates for community drug maintenance treatment, clean needle provision or exchange (2.93%, 673 participants) and peer education (4.83%, 1,109 participants) were strikingly low.

**Table 2:** HIV-related knowledge, behaviors and services for male STD clinics attendees in Southwest China, 2010–2022 (n,%)

Variable	Total	2010	2011	2012	2013	2014	2015	2016	2017	2018	2019	2020	2021	2022	Z value	*P* for trend
	*N* = 22,955	*N* = 1501	*N* = 1726	*N* = 1825	*N* = 1986	*N* = 2119	*N* = 2095	*N* = 2203	*N* = 1860	*N* = 1905	*N* = 1560	*N* = 1580	*N* = 1537	*N* = 1058		
**Had adequate HIV-related knowledge**																
No	1533	342	264	116	80	118	96	96	144	95	50	68	40	24		
	6.68%	22.78%	15.30%	6.36%	4.03%	5.57%	4.58%	4.36%	7.74%	4.99%	3.21%	4.30%	2.60%	2.27%		
Yes	21,422	1159	1462	1709	1906	2001	1999	2107	1716	1810	1510	1512	1497	1034	3.0	**0.002**
	93.32%	77.22%	84.70%	93.64%	95.97%	94.43%	95.42%	95.64%	92.26%	95.01%	96.79%	95.70%	97.40%	97.73%		
**Had visited FSWs in the last three months**																
No	16,170	1023	1398	1287	1843	1513	1204	1335	1259	1262	1079	1154	1120	693		
	70.44%	68.15%	81%	70.52%	92.80%	71.40%	57.47%	60.60%	67.69%	66.25%	69.17%	73.04%	72.87%	65.50%		
Yes	6785	478	328	538	143	606	891	868	601	643	481	426	417	365	0.4	0.700
	29.56%	31.85%	19%	29.48%	7.20%	28.60%	42.53%	39.40%	32.31%	33.75%	30.83%	26.96%	27.13%	34.50%		
**Had casual sex partner in the last three months**																
No	17,283	1014	1538	1692	1789	1686	1327	1300	1284	1455	990	1229	1130	849		
	75.29%	67.55%	89.11%	92.71%	90.08%	79.57%	63.34%	59.01%	69.03%	76.38%	63.46%	77.78%	73.52%	80.25%		
Yes	5672	487	188	133	197	433	768	903	576	450	570	351	407	209	0.4	0.700
	24.71%	32.45%	10.89%	7.29%	9.92%	20.43%	36.66%	40.99%	30.97%	23.62%	36.54%	22.22%	26.48%	19.75%		
**History of IDU**																
No	22,817	1474	1713	1817	1964	2103	2082	2189	1855	1904	1554	1568	1537	1057		
	99.40%	98.20%	99.25%	99.56%	98.89%	99.24%	99.38%	99.36%	99.73%	99.95%	99.62%	99.24%	100%	99.91%		
Yes	138	27	13	8	22	16	13	14	5	1	6	12	0	1	-2.0	**0.010**
	0.60%	1.80%	0.75%	0.44%	1.11%	0.76%	0.62%	0.64%	0.27%	0.05%	0.38%	0.76%	0.00%	0.09%		
**Had homosexual behavior**																
No	22,903	1498	1725	1825	1974	2118	2094	2196	1855	1897	1559	1573	1535	1054		
	99.77%	99.80%	99.94%	100%	99.40%	99.95%	99.95%	99.68%	99.73%	99.58%	99.94%	99.56%	99.87%	99.62%		
Yes	52	3	1	0	12	1	1	7	5	8	1	7	2	4	1.0	0.200
	0.23%	0.20%	0.06%	0%	0.60%	0.05%	0.05%	0.32%	0.27%	0.42%	0.06%	0.44%	0.13%	0.38%		
**History of STD**																
No	21,786	1069	1631	1746	1940	1964	2022	2169	1829	1833	1508	1541	1502	1032		
	94.91%	71.22%	94.50%	95.67%	97.68%	92.69%	96.52%	98.46%	98.33%	96.22%	96.67%	97.53%	97.72%	97.54%		
Yes	1169	432	95	79	46	155	73	34	31	72	52	39	35	26	-2.0	**0.030**
	5.09%	28.78%	5.50%	4.33%	2.32%	7.31%	3.48%	1.54%	1.67%	3.78%	3.33%	2.47%	2.28%	2.46%		
**Received service of CPD/VCT**																
No	7877	651	1001	1107	765	939	453	273	796	653	487	401	230	121		
	34.31%	43.37%	58%	60.66%	38.52%	44.31%	21.62%	12.39%	42.80%	34.28%	31.22%	25.38%	14.96%	11.44%		
Yes	15,078	850	725	718	1221	1180	1642	1930	1064	1252	1073	1179	1307	937	3.0	**0.004**
	65.69%	56.63%	42%	39.34%	61.48%	55.69%	78.38%	87.61%	57.20%	65.72%	68.78%	74.62%	85.04%	88.56%		
**Received service of CDMT/CNP**																
No	22,282	1235	1644	1802	1948	2083	2085	2181	1840	1773	1540	1559	1535	1057		
	97.07%	82.28%	95.25%	98.74%	98.09%	98.30%	99.52%	99%	98.92%	93.07%	98.72%	98.67%	99.87%	99.91%		
Yes	673	266	82	23	38	36	10	22	20	132	20	21	2	1	-2.0	**0.040**
	2.93%	17.72%	4.75%	1.26%	1.91%	1.70%	0.48%	1%	1.08%	6.93%	1.28%	1.33%	0.13%	0.09%		
**Received service of peer education**																
No	21,846	1284	1605	1775	1952	1885	2027	2175	1797	1724	1500	1541	1526	1055		
	95.17%	85.54%	92.99%	97.26%	98.29%	88.96%	96.75%	98.73%	96.61%	90.50%	96.15%	97.53%	99.28%	99.72%		
Yes	1109	217	121	50	34	234	68	28	63	181	60	39	11	3	-2.0	**0.030**
	4.83%	14.46%	7.01%	2.74%	1.71%	11.04%	3.25%	1.27%	3.39%	9.50%	3.85%	2.47%	0.72%	0.28%		

Note: Statistically significant P values are indicated in bold. FSWs, female sex workers; IDU, intravenous drug use; STD, sexually transmitted disease; CPD, condom promotion and distribution; VCT, HIV/AIDS voluntary counseling and testing; CDMT, community drug maintenance treatment; CNP, clean needle provision or exchange

**Fig. 1:**
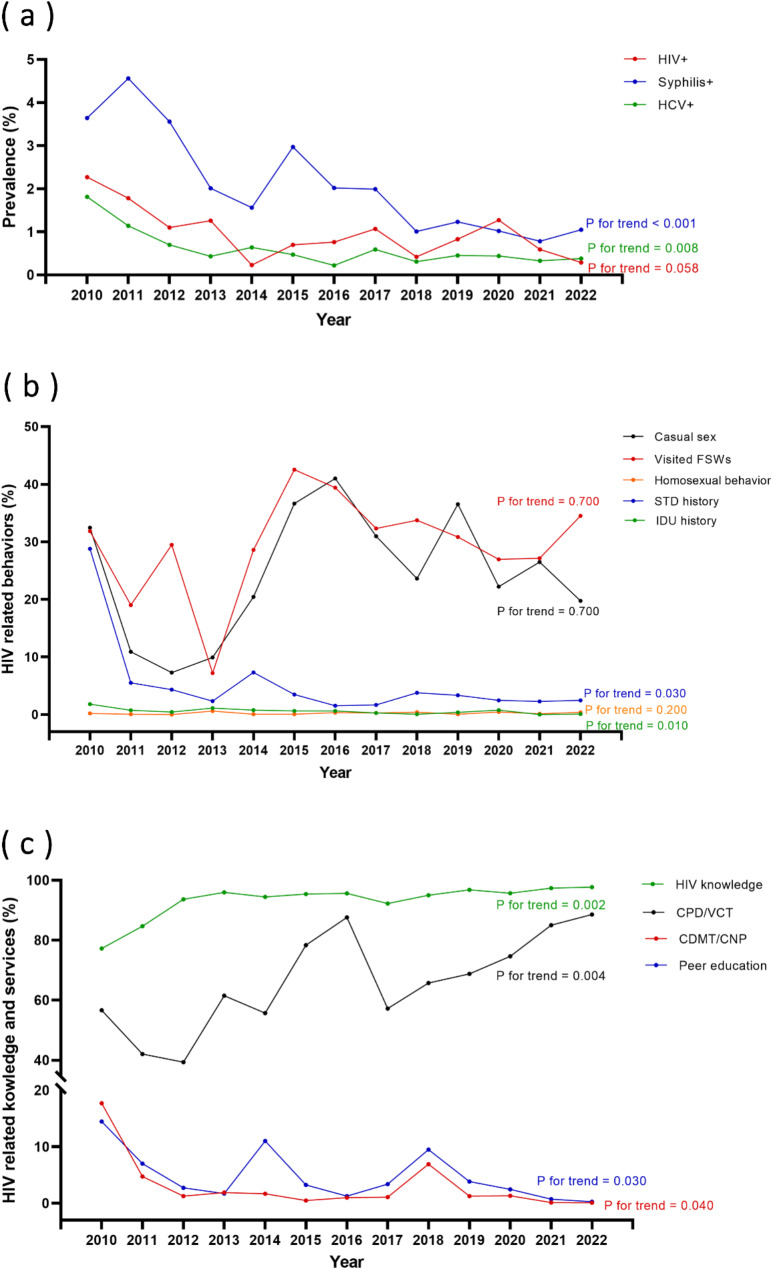
Trends in HIV, HCV, and syphilis prevalence, HIV-related behaviors, HIV-related knowledge and services among respondents, 2010–2022. (**a**) trends in HIV, HCV, and syphilis prevalence; (**b**) trends in HIV-related behaviors; (**c**) trends in HIV-related knowledge and services

Figure 1(b, c) illustrates trends observed during the study period. During the study, the proportion of participants with adequate HIV-related knowledge gradually increased (P for trend = 0.002). Conversely, the proportion of participants who reported engaging in commercial sex with FSWs and engaging in casual sex in the last three months remained relatively stable, showing no significant changes.

Furthermore, participants receiving condom promotion and distribution or HIV voluntary counseling and testing program services exhibited an upward trend (P for trend = 0.004). In contrast, the utilization of services for community drug maintenance treatment, clean needle provision or exchange (P for trend = 0.040), and peer education (P for trend = 0.030) showed a decreasing trend. Additionally, participants with a history of STD (P for trend = 0.030) and IDU exhibited a declining trend (P for trend = 0.010). In contrast, those with a history of homosexual behavior did not demonstrate a significant trend.

### Prevalence, trends of HIV, HCV, and syphilis

Table 3; Fig. 1(a) provided a comprehensive overview of the prevalence of HIV, syphilis, and HCV among the study participants. The overall prevalence of HIV stood at 0.98%. Over the study period from 2010 to 2022, the HIV prevalence decreased from 2.27% in 2010 to 0.29% in 2022, without a significant downward (P for trend = 0.058). The prevalence of syphilis among participants was 2.16%. Notably, there was a consistent decline in syphilis prevalence, with rates decreasing from 3.64% in 2010 to 1.05% in 2022 (P for trend < 0.001). The study also examined the prevalence of HCV, which was found to be 0.61%. Like syphilis, the HCV prevalence displayed a remarkable decreasing trend, declining from 1.81% in 2010 to 0.38% in 2022 (P for trend = 0.008).

**Table 3:** Prevalence of HIV, Syphilis, and HCV among study respondents in Southwest China, 2010–2022 (n, %)

Infection	Total	2010	2011	2012	2013	2014	2015	2016	2017	2018	2019	2020	2021	2022	Z value	*P* for trend
	(*N* = 23893)	(*N* = 1761)	(*N* = 2019)	(*N* = 1997)	(*N* = 2070)	(*N* = 2170)	(*N* = 2138)	(*N* = 2229)	(*N* = 1872)	(*N* = 1916)	(*N* = 1560)	(*N* = 1580)	(*N* = 1530)	(*N* = 1051)		
**HIV+**	234	40	36	22	26	5	15	17	20	8	13	20	9	3		
	0.98%	2.27%	1.78%	1.10%	1.26%	0.23%	0.70%	0.76%	1.07%	0.42%	0.83%	1.27%	0.59%	0.29%	-1.891	0.058
	(*N* = 22876)	(*N* = 1317)	(*N* = 1931)	(*N* = 1827)	(*N* = 1989)	(*N* = 2111)	(*N* = 2085)	(*N* = 2177)	(*N* = 1858)	(*N* = 1885)	(*N* = 1548)	(*N* = 1572)	(*N* = 1529)	(*N* = 1047)		
**Syphilis+**	494	48	88	65	40	33	62	44	37	19	19	16	12	11		
	2.16%	3.64%	4.56%	3.56%	2.01%	1.56%	2.97%	2.02%	1.99%	1.01%	1.23%	1.02%	0.78%	1.05%	-3.355	**< 0.001**
	(*N* = 23957)	(*N* = 1766)	(*N* = 2017)	(*N* = 2005)	(*N* = 2084)	(*N* = 2188)	(*N* = 2148)	(*N* = 2226)	(*N* = 1872)	(*N* = 1916)	(*N* = 1560)	(*N* = 1580)	(*N* = 1537)	(*N* = 1058)		
**HCV+**	147	32	23	14	9	14	10	5	11	6	7	7	5	4		
	0.61%	1.81%	1.14%	0.70%	0.43%	0.64%	0.47%	0.22%	0.59%	0.31%	0.45%	0.44%	0.33%	0.38%	-2.623	**0.008**

Note: Statistically significant P values are indicated in bold

### Factors associated with HIV, syphilis, and HCV infections

**Fig. 2:**
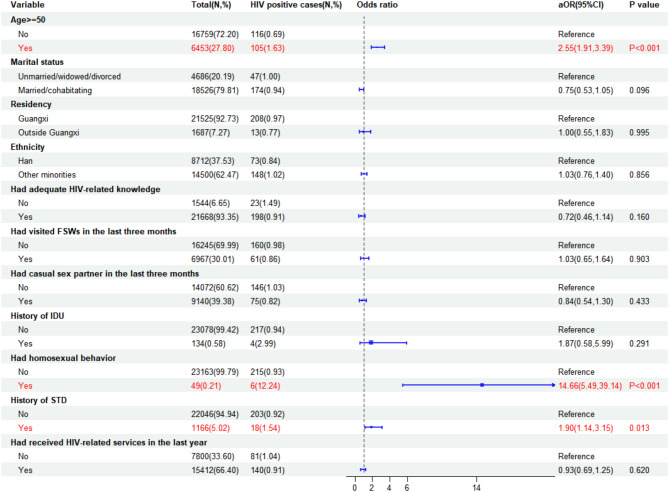
Correlates of HIV infection among male STD clinic attendees in Southwest China, 2010–2022

**Fig. 3:**
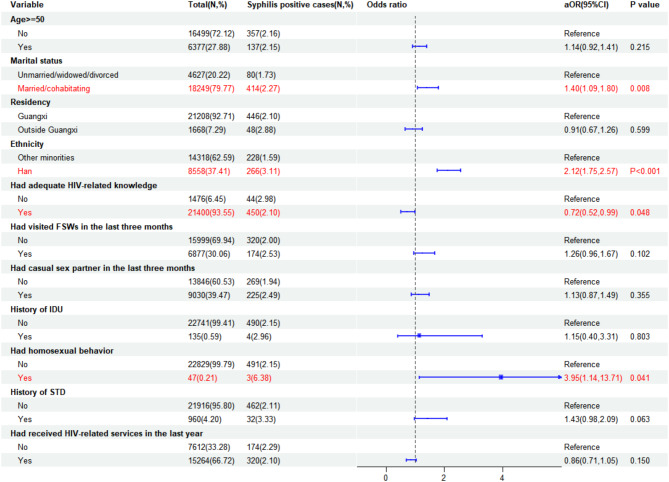
Correlates of syphilis infection among male STD clinic attendees in Southwest China, 2010–2022

**Fig. 4:**
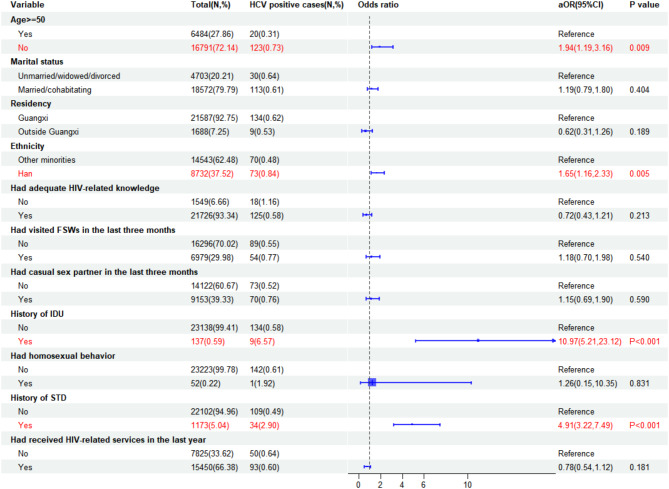
Correlates of HCV infection among male STD clinic attendees in Southwest China, 2010–2022

Figures 2, 3, and 4 showed the factors associated with HIV, syphilis, and HCV infections among male attendees at STD clinics. The prevalence of HIV infection was notably higher among male STD clinic attendees aged over 50 years old (aOR = 2.55, 95%CI: 1.91–3.39), those who reported homosexual behavior (aOR = 14.66, 95%CI: 5.49–39.14), and individuals with a history of STD (aOR = 1.90, 95%CI: 1.14–3.15).

For syphilis infection, several factors were identified. Being married or cohabiting (aOR = 1.40, 95% CI: 1.09–1.80) and of ethnic Han (aOR = 2.12, 95% CI: 1.75–2.57) were positively correlated with a higher likelihood of contracting syphilis. Additionally, individuals who reported engaging in homosexual behavior (aOR = 3.95, 95% CI: 1.14–13.71) were at an increased risk. However, having adequate HIV-related knowledge (aOR = 0.72, 95% CI: 0.52–0.99) was associated with a reduced likelihood of syphilis infection.

The analysis revealed that HCV infection was positively associated with various factors. Individuals aged less than 50 years (aOR = 1.94, 95% CI: 1.19–3.16) and of ethnic Han (aOR = 1.65, 95% CI: 1.16–2.33) were more likely to contract HCV. Furthermore, those with a history of intravenous drug use (IDU) (aOR = 10.97, 95% CI: 5.21–23.12) and a history of STD (aOR = 4.91, 95% CI: 3.22–7.49) exhibited a higher risk of HCV infection.

## Discussion

Our study marks the pioneering effort to document the province-wide prevalence of sexually transmitted infections (STIs) among male attendees at STD clinics in Southwest China. To our knowledge, this study is characterized by the most extensive sample size and the longest observational period, spanning 13 years of sentinel surveillance data. Notably, the HIV prevalence did not exhibit a significant declining trend. However, there were observed decreasing trends in the prevalence of syphilis and HCV infections. We identified that HIV, HCV, and syphilis infections were significantly associated with engaging in homosexual behavior, having a history of sexually transmitted infections (STIs), and intravenous drug use (IDU). These findings furnish critical insights into the prevalence and the risk factors underpinning HIV, syphilis, and HCV infections among male attendees at STD clinics, underscoring the paramount importance of devising tailored prevention and intervention strategies that take these specific risk factors into account.

In this study, we observed that the prevalence of HIV among male attendees at STD clinics in Southwest China was 0.98%, a figure closely in line with the finding of a similar study (0.7%) conducted in other regions of China [17]. Notably, the prevalence of HCV was substantially lower at 0.61% compared to a study conducted among STD clinic patients in Tennessee in 2016, where it was reported at 3.4% [29]. Similarly, the prevalence of syphilis (2.16%) was significantly lower than that reported in a previous study (11.9%) conducted between 2004 and 2006 in the same region [15]. Within this study, we identified decreasing trends in the prevalence of HCV and syphilis. These trends suggested that the strategies advocated by the Guangxi government to combat STIs, which encompass intensified testing, target intervention for various risk groups, and comprehensive sexual health education, have exerted a favorable impact in curbing the spread of these infections [30]. Our study also points to increasing awareness of HIV-related knowledge and services, along with the decreasing proportion of male clinic attendees with a history of drug use, as factors that support this explanation.

In contrast to a prior study that analyzed 13 years of data from 2004 to 2016, which predicted an escalation in HIV incidence [31], our study did not observe any upward trajectory in HIV prevalence. This suggested that the extensive efforts by the Chinese government to strengthen HIV control measures, including collecting and monitoring epidemiological data and adopting the World Health Organization’s 95-95-95 policy, have yielded positive outcomes in curbing the growth of the HIV epidemic. However, there was no statistically significant decreasing trend in HIV prevalence despite increased awareness of HIV-related knowledge and services. This may be attributed to the fact that certain high-risk behaviors may persist or even increase, which can counteract the benefits of increased awareness and prevention efforts. Therefore, it is now imperative to consider expanding or implementing routine opt-out HIV screening in healthcare settings. Special attention should be given to methods that better integrate HIV screening into the workflow of STD clinics. Moreover, developing locally tailored HIV testing programs, including initiatives to reach individuals in non-healthcare settings through strategies like HIV self-testing, is warranted. These measures will contribute to a more comprehensive and effective approach to HIV prevention and control.

Our study revealed a striking association between male STD clinic attendees who had engaged in homosexual behavior and their susceptibility to HIV and syphilis, with infection rates 14.66 and 3.95 times higher, respectively, compared to those who had never had homosexual experiences. This finding is in alignment with a similar study that reported a higher risk of HIV infection among individuals who engaged in homosexual or bisexual practices [17]. Furthermore, we identified a significant link between self-reported STIs in the past year and the risk of HIV and HCV infections. This can be attributed to the shared transmission routes and potential for co-infection among HIV, syphilis, HCV, and other STIs [32–34]. A study conducted among attendees of public STD clinics in Hong Kong also demonstrated a positive association between a history of STIs and HIV infection [16]. These findings underscore the critical importance of intensifying sexual health education initiatives, particularly with a specific focus on homosexual men. Promoting consistent condom usage and advocating for regular testing for HIV, syphilis, and other STIs within this demographic is of utmost significance. Additionally, enhancing comprehensive surveillance and screening for STIs should be part of an integrated approach to effectively address this public health challenge.

Furthermore, our study identified a higher prevalence of HIV among participants aged 50 years and above, in line with the observed increase in HIV infection among older males in Southwest China [35]. One plausible explanation is that older males may be more inclined to engage in risky sexual behaviors, leading to their higher HIV prevalence. Multiple factors likely influence this trend. For instance, experiences like divorce or widowhood might drive them to seek casual sex partners. Additionally, societal ageism might make them more vulnerable to engaging in sexual encounters with sex workers. Furthermore, a lack of HIV-related knowledge could result in a diminished sense of self-protection during sexual activity [36]. Therefore, it is crucial to strengthen sexual morality education within HIV prevention and control efforts. Moreover, particular attention should be directed towards HIV awareness and educational programs targeting older men living alone while emphasizing the importance of compassionate care. Public health personnel should also integrate HIV-related knowledge into various formats during awareness and education campaigns, ensuring its dissemination through diverse channels to continually enhance HIV prevention awareness among the elderly.

This study observed a higher prevalence of syphilis and HCV among male attendees at STD clinics who identified as Han ethnicity. It’s important to note that our surveyed area predominantly comprises individuals of Han ethnicity, which may contribute to these findings. In contrast to prior research suggesting a higher prevalence of syphilis among single participants [37], our study observed an increased susceptibility to syphilis among male outpatients who were married or cohabiting. This difference could be attributed to the lower condom use among married or cohabiting individuals, potentially contributing to an elevated syphilis incidence [38]. It is widely recognized that engaging in unprotected sexual intercourse constitutes a primary risk factor for STIs [39]. Additionally, in our univariable analysis (Supplementary Fig. 1), syphilis infection was associated with a history of commercial sex and casual sex among the overall population and married or cohabiting participants. At the same time, this association was not significant among unmarried, widowed, or divorced individuals. We hypothesize that married or cohabiting male outpatients may be more likely to have a history of commercial sex and casual sex, contributing to the higher prevalence of syphilis. This result emphasized the necessity of promoting the awareness of STIs-related knowledge, encouraging couples to undergo regular STD testing, and intensifying the promotion and distribution of condoms, including providing free or low-cost condoms, to encourage more individuals to use them during sexual intercourse.

Furthermore, our study indicated that male STD clinic attendees who had a history of IDU had a significantly increased risk of HCV infection. An existing excellent review has emphasized the close connection between IDU and HCV transmission [40]. Guangxi shares a 1020-kilometer borderline with Vietnam, and its proximity to the “Golden Triangle” has made it a significant route for drug trafficking [13]. In contrast to HIV, our study observed a higher likelihood of HCV infection among individuals under the age of 50. This differs from previous findings that suggested older age as a risk factor for HCV infection [41]. However, it is important to consider that, in the China–Vietnam border area, more than half (54.09%) of the drug users are young (aged 13–34 years) [42]. A multistage systematic review further estimated that 27.9% of people who inject drugs (PWID) globally are under 25 years of age [43]. Consistent with these findings, our study also observed a significantly higher proportion of individuals under 50 years reporting a history of intravenous drug use (IDU) (0.70%) compared to those aged 50 and above (0.32%) (Supplementary Table 1). This disparity likely contributed to the increased prevalence of HCV in the younger age group. Therefore, to reduce the transmission of HCV, it is crucial to implement the following measures. First, offer rehabilitation programs and addiction treatment services for individuals with a history of substance abuse to help them overcome addiction, reduce the need for injecting drugs, and thereby lower the risk of HCV transmission. Second, conduct public education campaigns to raise awareness about HCV, particularly the risks associated with intravenous drug use and unsafe injection practices. Third, widespread HCV screening and treatment services should be promoted to enable early diagnosis and treatment of infections, preventing further transmission.

This study had several limitations. The results did not suggest a causal relationship, and they were only applicable to males, not females. Firstly, it relied on self-reported data, which could potentially lead to underreporting of certain behaviors, such as having multiple sexual partners or engaging in homosexual behavior due to social stigma and discrimination. Additionally, self-reported data may be subject to recall bias or information bias, potentially influencing our analysis. Secondly, the relatively low number of positive cases may have constrained our ability to identify additional pertinent risk factors in the study. Thirdly, the number of participants surveyed varies from year to year, and the survey did not include an assessment of condom usage, a potentially crucial indicator of high-risk sexual behavior. Finally, we used HCV-positive-antibody testing to identify HCV infection. However, a positive antibody test cannot distinguish between an active infection and a resolved infection, which might have overestimated the prevalence of HCV.

This study was the first of its kind to assess the prevalence of HIV, HCV, and syphilis, as well as the associated risk factors, among a large sample of male STD clinic attendees in the cross-border region of Southwest China. Despite the previously mentioned limitations, the prevalence figures obtained in this extensive and consecutive study were based on sentinel surveillance data, and they may closely reflect the actual prevalence of these three STIs among male STD clinic attendees in this border area. Nevertheless, additional research focusing on the behavioral risk factors influencing the emergence and transmission of STIs in this population of Southwest China is warranted.

## Conclusions

This study, spanning 13 years in Southwest China, showed the relatively low prevalence of HIV, syphilis, and HCV among male STD clinic attendees, with decreasing trends for syphilis and HCV. The main risk factors for STIs infection included homosexual behavior, a history of STIs, and intravenous drug use. These findings highlight the importance of ongoing efforts to promote safe sexual practices, raise awareness, and improve access to preventive services. They also underscore the continued need for comprehensive sexual health programs to further reduce STIs incidence among male attendees at STD clinics.

## Electronic supplementary material

Below is the link to the electronic supplementary material.

Supplementary Material 1

## Data Availability

Due to ethical and legal considerations, the datasets generated and/or analyzed during this study are not publicly accessible. However, they are available from the corresponding author, Bingyu Liang, upon reasonable request.

## Declarations

### Clinical trial registration details

Clinical trial number: not applicable.

## Abbreviations

HIV: Human immunodeficiency virus
HCV: Hepatitis C virus
STIs: Sexually transmitted infections
STD: Sexually transmitted disease
FSWs: Female sex workers
IDU: Intravenous drug use
CPD: Condom promotion and distribution
VCT: HIV/AIDS voluntary counseling and testing
CDMT: Community drug maintenance treatment
CNP: Clean needle provision or exchange
