# Supplementary material for: Prevalence, trends, and correlates of HIV, syphilis, and HCV infections among male attendees at STD clinics in Southwest China: a 13-year cross-sectional study (2010–2022)
Source: BMC Infect Dis. 2025 Feb 12;25:205. doi: 10.1186/s12879-025-10571-9 (PMC11817693; doi:10.1186/s12879-025-10571-9)
Supplement: Supplementary file 1 — Supplementary Material 1 [file 12879_2025_10571_MOESM1_ESM.pdf]

Supplementary Material

Title: Prevalence, trends, and correlates of HIV, syphilis, and HCV infections among male attendees at STD clinics in Southwest China: A 13-year cross-sectional study(2010-2022)

Content

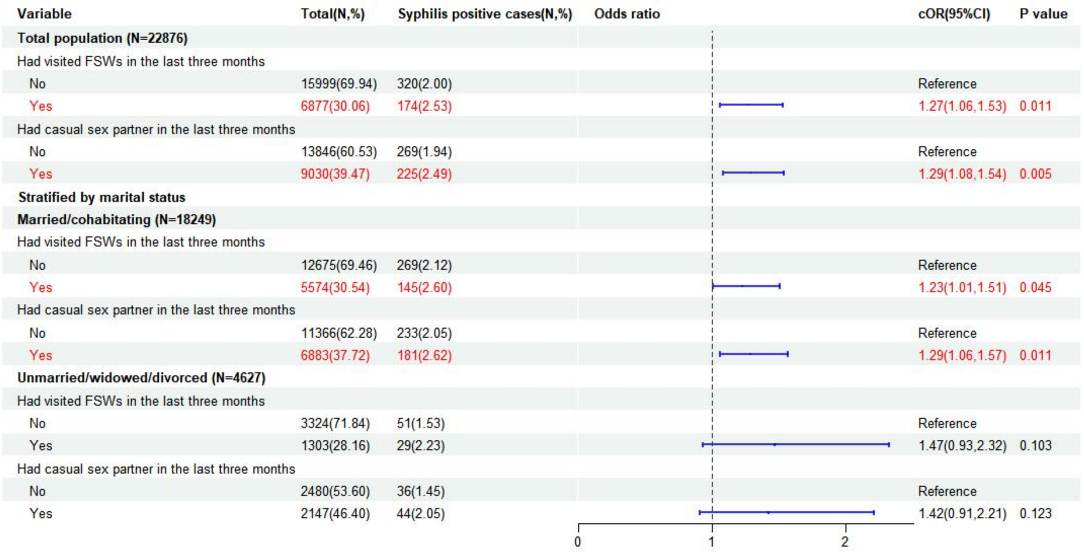

Supplementary Figure 1. Association of syphilis infection and commercial sex or casual sex, stratified by marital status.

Supplementary Table 1. Number(%) of participants with a history of IDU by age.

Age	History of IDU		Total	P
	No	Yes		
<50	16675(99.30%	116(0.70%)	16791	
>=50	6463(99.68%)	21(0.32%)	6484	0.001
	23138	137	23275	
